# Temporal interaction information and laser evoked responses: preliminary results in fibromyalgia patients with small fibers pathology

**DOI:** 10.3389/fnetp.2025.1592518

**Published:** 2025-09-01

**Authors:** Livio Clemente, Marianna La Rocca, Sebastiano Stramaglia, Daniele Marinazzo, Raffaella Lombardi, Giuseppe Lauria, Marina de Tommaso

**Affiliations:** ^1^ Neurophysiopathology Unit, DiBRain Department Bari Aldo Moro University, Bari, Italy; ^2^ Physic Department, Bari Aldo Moro University, Bari, Italy; ^3^ Department of Data Analysis Faculty of Psychological and Educational Sciences, Ghent, Belgium; ^4^ IRCCS Istituto Neurologico “Carlo Besta”Dipartimento Neuroscienze Cliniche, Milano, Italy

**Keywords:** laser evoked potentials, pain rating, fibromyalgia, small fibers pathology, temporal interaction information, network physiology

## Abstract

**Objectives:**

We propose a novel application of higher-order information-theoretic measures to assess the temporal interaction information (TII) between laser-evoked potentials (LEPs) and individual pain ratings in healthy subjects and patients with fibromyalgia (FM) affected by small fiber pathology.

**Methods:**

Seventy-nine FM patients, categorized into three groups based on skin biopsy findings (normal innervation n 19, proximal denervation n 53, and both proximal and distal denervation n 7), and 14 control subjects were studied. We used cluster-based permutation tests (p < 0.05) to identify significant clusters of TII between cortical components recorded using a 64-channel electroencephalography (EEG) system - with focus on the Cz electrode - and subjective pain ratings to quantify synergy or redundancy between the LEP signal time points and Visual Analog Scale (VAS).

**Results:**

Control subjects generally exhibited synergy clusters corresponding to N2 and P2 peaks, whereas patients with fibromyalgia (FM), particularly those with distal denervation, exhibited increased redundancy and decreased synergy.

**Conclusion:**

Patients with FM and small fiber pathology exhibited an alteration in higher-order integration mechanisms due to a complex interaction between cortical processing of pain and denervation of nociceptive fibers.

**Significance:**

These findings highlight the potential of TII in elucidating the complex interplay between peripheral nerve integrity and central sensitization in FM and other chronic pain disorders.

## Highlights

• Temporal interaction Information between laser evoked potentials and pain rating illustrates the complexity of pain processing.
• Patients with fibromyalgia and small fibers pathology showed decreased synergy between vertex complex time points and pain rating.
• Temporal Interaction Information could explain the complex interplay between peripheral and central factors in chronic pain.

## 1 Introduction

Fibromyalgia (FM) is a chronic pain syndrome defined by the presence of widespread musculoskeletal pain in multiple areas of the body for at least 3 months (Wolfe et al., 2010; 2016). The FM diagnosis is confirmed by its association with fatigue, poor sleep, impaired cognition and other symptoms such as headache, abdominal pain and depression (Wolfe et al., 2016).

Central sensitization phenomena are the basic mechanism in FM, which according to the International Association for Pain Study (IASP) meets the definition of nociplastic pain (Arendt-Nielsen et al., 2015). Increased sensitivity to nociceptive stimuli, called hyperalgesia, is a central phenomenon in FM that is mainly due to impaired pain processing at the central level, although ample evidence points to the coexistence of peripheral small fiber neuropathy (Sluka and Clauw, 2016). FM syndrome is the result of a complex interplay of factors favoring central sensitization, such as sleep disorders and psychopathological profile, and small fiber neuropathy (De Tommaso et al., 2022). Instrumental assessment of chronic pain symptoms is challenging, especially in a complex syndrome such as FM, where dysfunction of central and peripheral nociceptive pathways appears to coexist. (De Tommaso et al., 2022; Devigili et al, 2023). Laser-evoked potentials are event-related responses obtained by the selective stimulation of nociceptive afferents, mainly adelta fibers (Hubli and Leone, 2024). While the first studies of these event-related responses indicated a strict correspondence between the amplitude of the late central complex, namely, N2P2, and the subjective perception of pain (Carmon et al., 1980), this strict association was discussed in terms of the expression of salience of the painful stimuli and the resulting defensive response more than the individual assessment of their intensity (Moayedi et al., 2015). The N2P2 complex is an evoked potential recorded by EEG at the vertex, characterised by a negative deflection (N2) followed by a positive deflection (P2) (Babiloni et al., 2008). The N2, peaking at 200–280 ms, is mainly generated in the anterior cingulate cortex (ACC), while the P2, peaking at 300–370 ms, involves the ACC and the secondary somatosensory cortex (SII). The most common measurement is the peak-to-peak amplitude between N2 and P2 recorded on Cz (Valeriani et al., 2000). In FM, the subjective perception of laser stimuli tended to increase compared to controls, regardless of the amplitude of the LEPs, which is a complex expression between the degree of denervation of the small fibers and the tendency to emphasize the painful input at the cortical level under repeated stimulation, i.e., reduced habituation (Vecchio et al., 2020; 2022).

The search for a measure of subjective pain perception could expand our knowledge of the complex mechanisms of pain processing in normal and pathological conditions. With this in mind, we aimed to perform a novel analysis of LEPs consisting of an integrated approach to temporal representation of the LEP vertex complex and individual pain perception in normal subjects and FM patients affected by different degrees of small fiber impairment.

The use of information theory makes it possible to identify higher-order relationships that cannot be captured by first-order connectivity analyses.

Information theory has been used extensively in neuroscience to understand the transmission and processing of information in complex brain networks and to describe connectivity dynamics in a quantitative and rigorous way (Borst and Theunissen, 1999; Cover and Thomas, 1991; 2006). Interaction information (II), an extension of mutual information (MI), is a particularly useful metric for assessing the dependency between two variables. It allows us to explore how the shared information is driven by the interaction of additional components of the neural system, providing a more comprehensive measure of the complex dynamics of the brain (Lizier et al., 2018; Stramaglia et al., 2021).

Recent studies have shown that analyzing higher-order interactions can reveal information not visible with traditional methods of first-order connectivity, improving our ability to understand potential changes in brain connectivity (Barrett, 2015; Ince et al., 2017). We therefore wanted to test the hypothesis that the implementation of a higher order temporal interaction involving three variables—namely, the EEG amplitude at each point in the −100 600 msec time frame expressed in x-y coordinates (each point on the x-axis with each point on the y-axis) and the Visual Analog Scale (VAS) value—could reveal a close relationship between ERP and subjective pain perception. We investigated possible differences between FM patients and control subjects and also determined the possible effects of the different types of small fiber impairments in the FM population as determined by skin biopsy. This analytic framework can be considered within the emerging field of Network Physiology, which focuses on the dynamic interactions between organ systems and their temporal coordination across multiple scales (Ivanov and Bartsch, 2014). In this scenario, the brain is conceptualised as a dynamic node embedded within a broader physiological network, wherein transient neural interactions contribute to systemic regulation (Ivanov, 2021). The application of Temporal Interaction Information (TII), an application of the II to the time domain that assesses how a specific stimulus feature is similarly represented across neural responses at different time points (Ravijts, 2019), to LEPs represents a novel contribution to this field, offering a tool to quantify functional integration across time within a single physiological system, the CNS, which complements previous work on cross-system coordination (Bartsch et al., 2015).

## 2 Materials and methods

### 2.1 Subjects

We analysed temporal interaction information in a subgroup of FM patients previously described in a study by Vecchio et al. (2020). This study included data from 81 patients consecutively recruited for skin biopsy and LEP analysis between January and December 2017. A total of 79 FM patients and 14 healthy controls were eligible for analysis in the present study. The control group consisted of healthy subjects aged between 18 and 75 years, who met the same inclusion and exclusion criteria applied to FM patients in Vecchio et al. (2022), with the only difference being the absence of a diagnosis of fibromyalgia. Exclusion criteria included education below 8 years and any cause of peripheral or central nervous system diseases (including spinal cord diseases and radiculopathies), diabetes, active thyroid insufficiency, renal failure, active autoimmune diseases and/or inflammatory arthritis, systemic connective tissue disease, psychiatric conditions other than anxiety and depression disorders according to the DSM-5, active malignancies or history of cancer, use of drugs acting on the central nervous system, and chronic opioid therapy. The FM group was divided into three subgroups based on the results of the skin biopsy: subjects with normal biopsy findings (IENFD-FMN, n. 19), those with proximal denervation (IENFD-FMP, n. 53) and those with both proximal and distal denervation (IENFD-FMD n. 7) as 1 patient in IENFD-FMP group and 1 patient in IENFD-FMD group presented with artifacts on Cz channel. The actual EEG analysis was based on data collected within the experimental protocol described in detail in Vecchio et al. (2020) and was approved by the Ethics Committee of the General Hospital of Bari according to the guidelines of the Declaration of Helsinki. An information letter about the additional analysis was sent to the same ethics committee, while the patients were informed about the possible use of EEG and clinical and skin biopsy data for research purposes.

As reported in detail in Vecchio et al. (2020), the following inclusion criteria were used in this study: 1) a diagnosis of fibromyalgia (FM) based on the criteria established by the American College of Rheumatology (ACR) in 1990 and updated in 2010 (Wolfe et al., 1990; 2010) and 2) an age between 18 and 75 years. The exclusion criteria were as follows: 1) the presence of peripheral nervous system (PNS) or central nervous system (CNS) disease, including spinal cord and radiculopathy, diabetes, active thyroid insufficiency, renal insufficiency, active autoimmune disease, inflammatory arthritis, systemic connective tissue disease, DSM-V psychiatric disorders, history of cancer, and use of medications affecting the CNS or chronic opiate therapy were exclusion factors. Patients were invited to start antiepileptic or antidepressant drug therapy only after a clinical, neurophysiological and skin biopsy examination. To avoid effects on LEP amplitudes, patients taking analgesics were asked to discontinue them at least 24 h prior to enrollment (Truini et al., 2010). All participants gave informed consent before participating in the study.

### 2.2 Laser evoked potentials

The laser stimulation procedure, performed with a CO_2_ laser (wavelength of 10.6 mm, beam diameter of 2 mm, and pulse duration of 30 ms). A total of 30 pulses were administered at each stimulation site, with an intensity set at 1.5 W above the individual pain threshold and a 10-s interstimulus interval. To prevent skin injury, fatigue, or nociceptor sensitization, the position of the laser beam was shifted after each pulse. A 5-min interval was maintained between stimulation blocks. The stimulation sequence (dorsal surface of the right hand, distal thigh, dorsal foot) was randomized across participants and, at the end of each series, perceived pain intensity was rated using a visual analog scale (VAS) from 0 to 100. EEG was recorded continuously during each stimulation block using a 64-channel system. The EEG recording and signal preprocessing methods are described in detail in the Supplementary Material.

### 2.3 Skin biopsy

The Supplementary Material provide a comprehensive overview of the skin biopsies, detailing the methodologies employed for sampling and the analysis of intraepidermal nerve fiber density (IENFD). Additionally, the Supplementary Material delineate the criteria adopted to subdivide patients according to the presence of any proximal and/or distal denervation. (Supplementary Appendix SA1; Lauria et al, 2010; Devigili et al, 2008).

### 2.4 Information-theoretical quantity calculation

The trials were summarized to obtain a more accurate estimate of the temporal information related to the LEPs modulated by the pain perception reported via the VAS. The analysis of temporal interactions was performed at the Cz electrode, which is typically associated with the expression of vertex LEPs (Treede et al., 2003). The EEG data were transformed using normalization by a Gaussian copula as suggested by Ince et al. (2017). This approach enabled the analytical computation of entropy in the case of Gaussian distributions, and it served to reduce the bias typically introduced by non-parametric entropy estimators, especially in limited-sample conditions (Xiong et al., 2017). However, this transformation attenuates amplitude-related information in the EEG signal, which may carry physiological relevance. The present study acknowledges this limitation and interprets its findings in light of this trade-off.

#### 2.4.1 Mutual information

Mutual information (MI) is a statistical measure that quantifies the dependence between two random variables. It provides an estimate of the reduction in uncertainty associated with one variable when the other is known. MI is an extremely versatile approach as it does not require any assumptions about the distributions of the variables or the nature (linear or non-linear) of the relationship between them. This makes it possible to capture both complex non-linear and non-monotonic effects (Cover and Thomas, 1991; 2006; Latham and Roudi, 2009). From a mathematical perspective, the MI between two random variables can be defined using three equivalent formulations based on entropy differences (Ince et al., 2017):

$$I\left(X;Y\right)=H\left(Y\right)-H\left(X\right)$$

$$=H\left(X\right)-H\left(Y\right)$$

$$=H\left(X\right)+H\left(Y\right)-H\left(X,Y\right)$$

where I represents Mutual Information, H(X) represents the marginal entropy of X, defined as the overall uncertainty associated with variable X taken individually, while H(Y) indicates the marginal entropy of Y, defined as the uncertainty of Y considered alone. The quantities H(Y∣X) and H (X∣Y) express the conditional entropy, thereby indicating the amount of uncertainty that remains about Y once X is known, or about X once Y is known. Finally, H (X,Y) represents the joint entropy of the two variables.

Each of these formulations offers a different perspective on the nature of the information shared between X and Y. The first expression, I (X; Y) = H(Y)-H(Y∣X), indicates that MI quantifies the average reduction in uncertainty about the variable Y once the value of X is known. This means that the acquisition of knowledge about X enables a more accurate prediction of Y and thus reduces the associated uncertainty. The second expression, I (X; Y) = H(X)-H (X∣Y), demonstrates the symmetry of MI, as the reduction in uncertainty is reciprocal. Mutual information (MI) quantifies both the reduction in uncertainty about Y through knowledge of X and, conversely, the reduction in uncertainty about X through knowledge of Y. The third expression, I (X; Y) = H(X)+H(Y)-H (X,Y), describes the difference between the total entropy of the two variables considered separately and the entropy of their joint distribution. In practice, this formulation represents the difference between a model in which X and Y are statistically independent and the actual joint distribution, thus enabling the extent of dependence between the two variables to be quantified.

In the present study, we used MI to assess the amount of information exchanged between the time of display of vertex LEPs and perceived pain on the VAS scale. This approach provides a quantitative measure of the link between the neural activity elicited by the laser stimulation and the subjective pain experience of the subjects, thus clarifying the degree of connection between the cortical response and the perception of pain. We looked at the overall average obtained in each group for the three stimulation sites.

#### 2.4.2 Temporal interaction information

In information theory, interaction information (II) is a higher-order measure that describes the relationships between three or more random variables. It is calculated by evaluating the amount of shared information between a target variable and two source variables. The interaction information between three random variables, X1, X2 and Y, can be expressed mathematically as follows:

$$I\left(X_{1;}X_{2};Y\right)=I\left(X_{1},X_{2};Y\right)\;‐\;I\left(X_{1};Y\right)\;‐\;I\left(X_{2};Y\right)$$

In the equation, the joint mutual information (JMI) I (X1,X2; Y) describes the total information that the two source variables X1 and X2 share with the target variable Y (Faes et al., 2016). In other words, it quantifies the knowledge that can be derived about Y when both source variables are considered together. Conversely, the quantities I (X1; Y) and I (X2; Y) represent the information that each individual source variable can provide about the target variable Y when considered separately. A positive value of II indicates a synergy effect, i.e., that the combination of responses provides more information about the target than each individual variable would. A negative value, on the other hand, indicates a redundant effect, which means that the source variables provide similar information about the target variable.

Temporal Interaction Information (TII) can be defined as an extension of classical interaction information applied to the temporal domain. The purpose of this extension is to allow the evaluation of how pairs of time points (in this case X_1_ and X_2_, during an EEG recording) jointly contribute to explaining the variability of behavioural responses (Y). In summary, TII is a quantitative metric that calculates the similarity of information regarding a specific stimulus feature across different time points within an observed signal. This measure enables the discernment of whether the combination of X_1_ and X_2_ provides redundant or synergistic information about Y, thus providing a temporal map of the interactions between brain components relevant to pain perception. In our sample TII was examined to assess the interconnectivity between the neuronal responses of LEPs measured at different time points and pain perception assessed with the VAS.

### 2.5 Statistical analyses

Features of LEPs, including VAS score, were evaluated by ANOVA with groups as conditions.

The analysis of interaction information (II) was conducted independently for each body site (hand, knee, foot) and across the four participant groups (controls, IENFD-FMN, IENFD-FMP, IENFD-FMD), with a focus on the Cz electrode, as this site optimally captures the N2P2 complex of the LEP. The computation considered a 700 millisecond time window, ranging from −100 milliseconds prior to the stimulus to 600 milliseconds post-stimulus. Within the proposed framework, two variables corresponded to LEP amplitudes at two distinct time points within the selected epoch, while the third variable was the VAS score from the same trial. The analysis targeted the largest clusters, particularly those that overlapped with, or occurred in close temporal proximity to, the N2 and/or P2 components. These components are key markers of nociceptive cortical processing at Cz, although TII was computed over the entire epoch.

A cluster permutation test was used to assess the statistical significance of the information-theoretic variables. This approach corrects for multiple testing across all possible time combinations while maintaining statistical significance, as described by Maris and Oostenveld (2007) and Pernet et al. (2015). The null distribution maps were subjected to a thresholding procedure (pre-cluster threshold: p < 0.05), in accordance with the pixel-based nonparametric thresholding method described by Cohen (2015). Subsequently, the largest clusters that exceeded the threshold were collected to create a maximum reference distribution. This distribution was then used to apply a threshold to the clusters observed in the empirical dataset, with a cluster threshold of p < 0.05. Cluster size was defined as the number of significant time pairs within a cluster, while cluster mass was calculated as the sum of the associated statistical values (e.g., t-values) across those time pairs. It is important to note that a significant cluster represents a contiguous set of time point pairs (T1, T2) in the LEP waveform whose joint interaction with the VAS score is statistically significant. This indicates temporally meaningful relationships between cortical activity and perceived pain.

## 3 Results

The amplitude and latency of vertex LEPs were similar between controls and all FM patients and between FM subgroups. The pain threshold was similar between groups, VAS scores were on average higher in patients’ groups, but they were quite variable determining not statistical significance (ANOVA with VAS at hand as variable and group as factor p 0.55; VAS at knee p 0.61; at foot 0.71), (Table 1). We did not find a relation between the pain rating and the amplitude of the N2 and P2 peaks in any of the groups considered.

**TABLE 1:** Mean ± standard deviation values of latency and amplitude of vertex N2P2 complex and pain rating measured in 0–100 VAS in controls (n° 14) and FM patients with normal intraepidermal nerve fibers density (IEFND-FMN), proximal denervation (IEFND-FMP) and proximal and distal denervation (IEFND-FMD).

Group	Age mean	District	VAS mean (sd)	N2 latency (ms)	N2 amplitude (u)	P2 latency (ms)	P2 amplitude (u)
Controls N°14 (12 f)	33,42 (±13,27)	Hand	51 (±10,73)	219	−7,23	363	17,51
		Kneel	57,78 (±21,93)	206	−7,86	355	14,612,023
		Foot	61,93 (±15,85)	233	−4,79	384	13,91
IEFND-FMN N° 19 (16 f)	43,05 (±12,37)	Hand	56,37 (±22,21)	222	−6,81	349	12,78
		Kneel	67,05 (±21,34)	207	−5,57	346	13,8
		Foot	62,68 (±21,24)	234	−5,42	376	9,67
IEFND-FMP N° 53 (45 f)	52,75 (±8,31)	Hand	54,34 (±20,92)	218	−6,75	347	9,83
		Kneel	64,24 (±23,2)	213	−5,8	356	10,1
		Foot	65,49 (±25,95)	230	−5,5	370	6,96
IEFND-FMD N° 7 (6 f)	41,71 (±10,75)	Hand	63,71 (±15,4)	231	−5,27	347	10,11
		Kneel	69 (±17,43)	209	−9,48	337	8,11
		Foot	66,86 (±21,74)	226	−5,32	365	6,2

### 3.1 Within subjects comparison

The aim of the within-analysis was to identify specific and consistent interactions between the temporal representation of LEPs and VAS scores in each group. This approach allows the investigation of the presence of synergy and redundancy patterns that may serve as distinctive markers of the nervous system’s response to somatosensory stimuli in relation to perceived pain (as measured by the VAS).

The analysis of the interaction information (II) showed the patterns of temporal interactions for each body district (hand, knee, foot) in the four groups of participants (controls, IENFD-FMN, IENFD-FMP, IENFD-FMD). The focus was on the largest clusters and those associated with or occurring in close proximity to the N2 and/or P2 components. The colormaps in the figures represent the magnitude of TII values across time points. Significant clusters (p < 0.05) identified through the cluster-based permutation test are outlined to indicate statistically meaningful regions.

**FIGURE 1:**
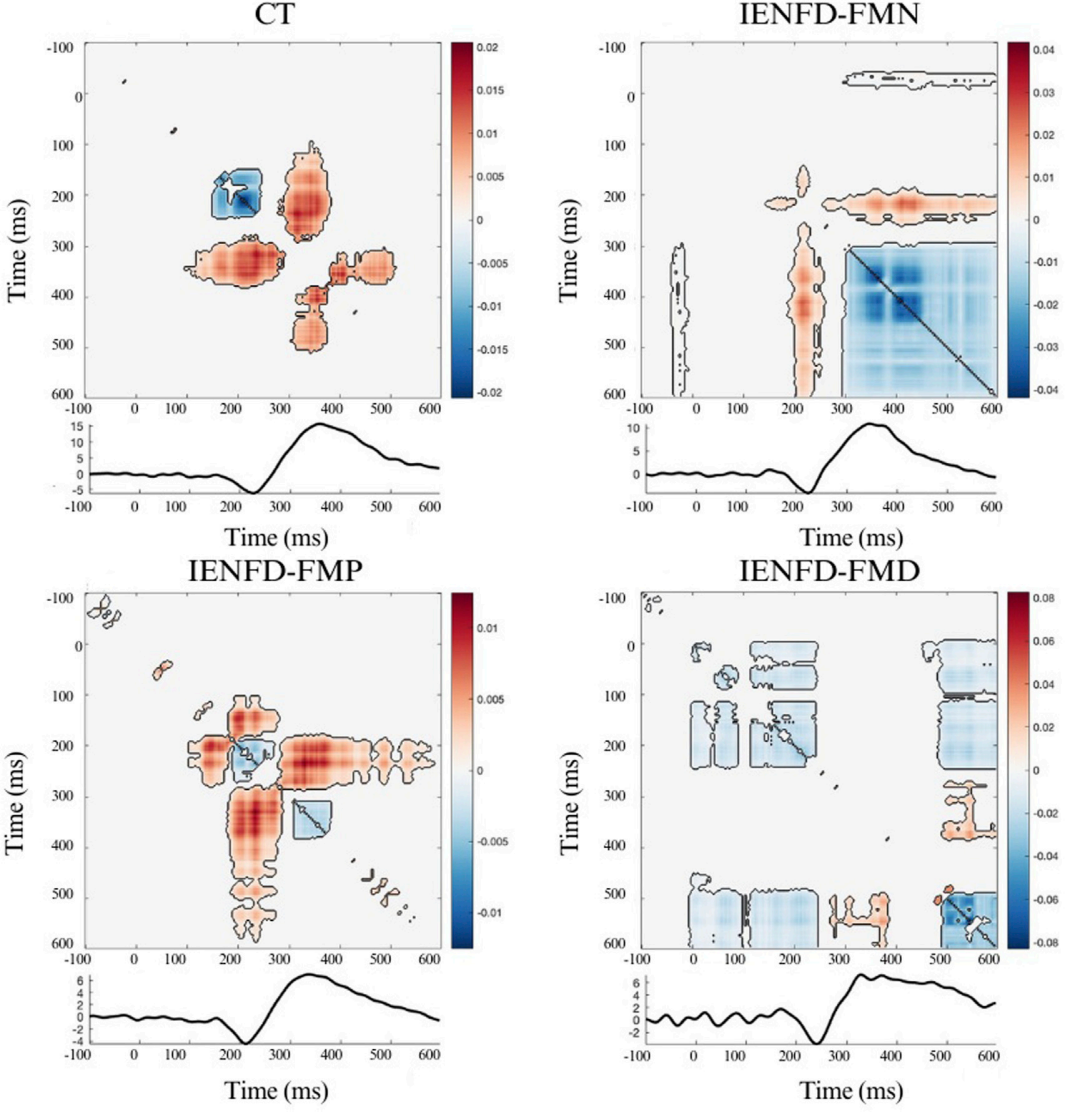
Nonparametric cluster-based permutation analysis of Temporal Interaction Information and the LEP wave in the Hand site, divided by group. The colormap shows the magnitude of TII across time points; positive values (warm colors) represent synergy, and negative values (cool colors) represent redundancy. Significant clusters (p < 0.05) are indicated by black contours.

Significant clusters were observed in the hand district in all four groups (Figure 1; Table 2). The controls showed three significant clusters, one of which showed a strong synergistic effect (cluster_mass = 3.22, cluster_size = 427) that occurred near the N2 and P2 components. FM patients with normal skin biopsy (IENFD-FMN) showed two synergy clusters and one large redundancy cluster (cluster_mass = −40.54, cluster_size = 2944) that overlapped with the N2 and P2 peaks. The IENF-FMP subgroup showed a synergistic effect with a cluster mass of 5.82 (cluster size = 1500). The IENF-FMD subgroup showed a lack of synergistic effect and several clusters with redundancy, including a cluster with a mass of −15.01 (cluster sie = 1018).

**TABLE 2:** Clusters size and weight of the nonparametric cluster-based permutation analysis within group and district.

Group	District	T1_min (ms)	T1_max (ms)	T2_min (ms)	T2_max (ms)	Cluster_mass	Cluster_size
Controls	Hand	150	232	165	244	−2.22	233
		95	291	279	381	7.10	847
		302	384	377	510	3.22	427
	Kneel	72	154	146	169	0.83	75
	Foot	44	68	64	83	0.29	25
		478	506	494	525	0.38	34
IENFD-FMN	Hand	−41	−10	294	596	−0.05	470
		142	201	205	228	0.42	69
		185	259	255	596	9.03	1021
		294	596	310	596	−40.54	2944
	Kneel	72	283	287	400	5.67	1145
		107	154	162	271	−1.377	297
		111	138	118	154	−0.27	44
		162	216	173	271	−1.19	217
	Foot	177	337	341	482	6.65	1169
		185	302	494	584	1.46	351
		388	431	400	455	−0.87	113
IENFD-FMP	Hand	103	236	181	279	1.16	539
		177	287	275	584	5.82	1500
		306	369	310	381	−0.47	159
	Kneel	21	107	271	463	−0.91	803
		25	72	44	103	−0.3	144
		142	162	146	177	−0.1	33
		271	467	537	596	1.79	720
		517	545	537	572	0.07	27
	Foot	29	107	244	334	0.67	378
		115	244	232	341	2.5	674
		232	349	237	588	6.88	1529
		248	302	255	314	−0.5	130
		373	404	381	431	−0.22	79
IENFD-FMD	Hand	−6	95	451	596	−8.74	821
		−2	87	115	244	−8.01	671
		44	75	52	87	−0.75	43
		103	244	482	596	−15.01	1018
		115	220	150	240	−7.13	433
		271	384	490	596	5.3	310
		490	592	502	596	−11.14	338
	Kneel	122	271	271	345	9.36	584
		154	216	169	236	−4.83	188
	Foot	424	451	431	455	−0.6	27
		517	592	549	596	−2.27	95

Note: T1_min and T1_max indicate the earliest and latest time points (in ms) of the first-time dimension of the significant cluster; T2_min and T2_max indicate the earliest and latest time points (in ms) of the second-time dimension. These values represent the temporal coordinates in the T1–T2 domain where the interaction information between two LEP, time points and the VAS score exceeded the statistical threshold.

**FIGURE 2:**
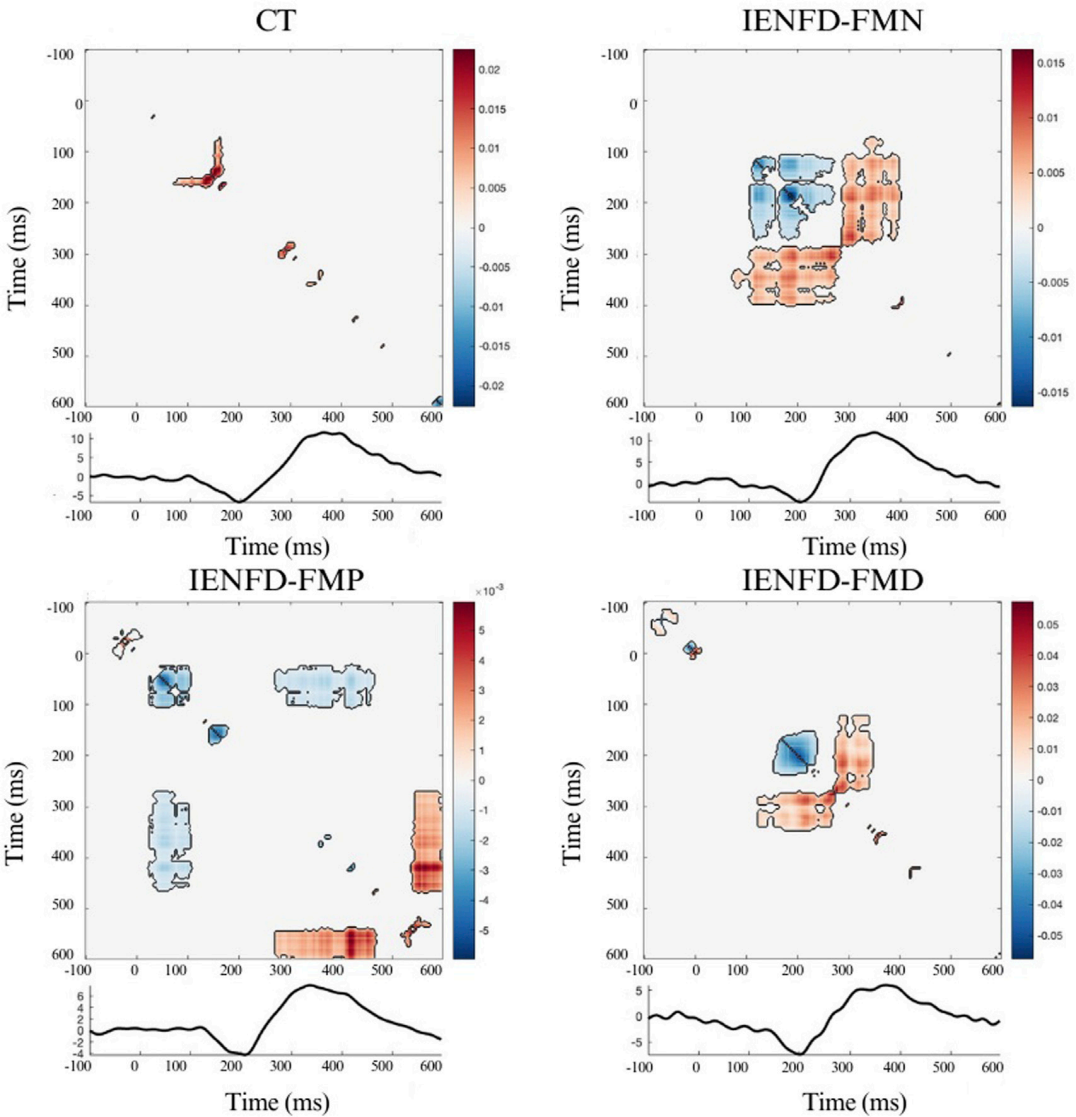
Nonparametric cluster-based permutation analysis of Temporal Interaction Information and the LEP wave in the Kneel site, divided into groups. The colormap shows the magnitude of TII across time points; positive values (warm colors) represent synergy, and negative values (cool colors) represent redundancy. Significant clusters (p < 0.05) are indicated by black contours.

In the knee region (Figure 2; Table 2), the control group showed a cluster of synergy consistent with the N2 wave, while patients with fibromyalgia (FM) showed clusters of synergy and redundancy in the time window of N2 and P2. In patients with proximal denervation, the temporal relationships were quite different and more scattered. In particular, patients with normal biopsy findings (IENF-FMN) showed multiple clusters, one of which covered a wide time window (cluster_mass = 5.67, cluster_size = 1145). A relatively small cluster was identified for the IENF-FMP subgroup (cluster_mass = −0.91, cluster size = 803), while the IENF-FMD subgroup showed a large positive cluster (cluster_mass = 9.36, cluster size = 584), which coincided with the N2 peak and partly with the P2 peaks.

**FIGURE 3:**
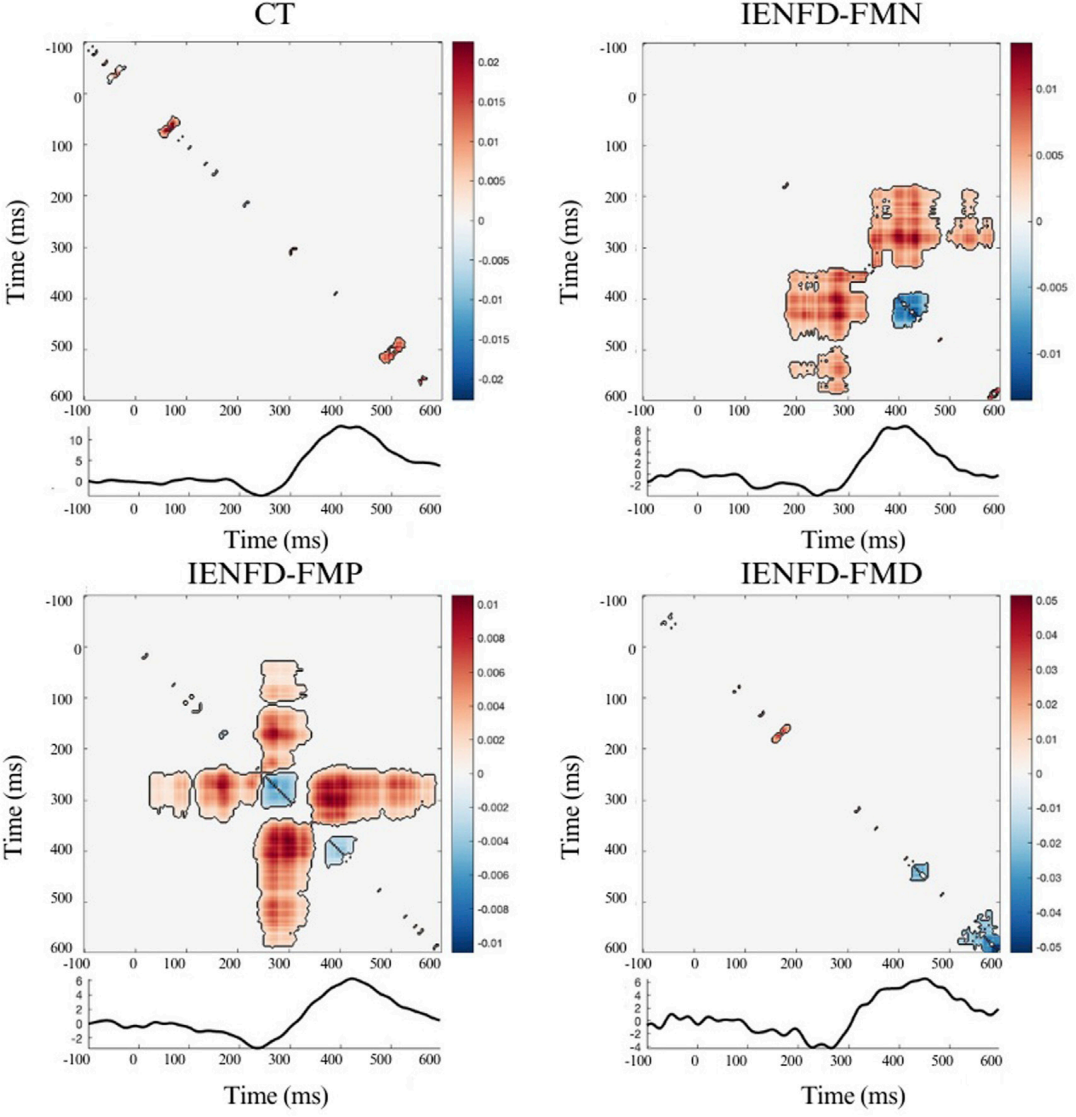
Nonparametric cluster-based permutation analysis of Temporal Interaction Information and the LEP wave in the Foot site, divided by group. The colormap shows the magnitude of TII across time points; positive values (warm colors) represent synergy, and negative values (cool colors) represent redundancy. Significant clusters (p < 0.05) are indicated by black contours.

Similar to the knee site, the foot site showed a limited temporal interaction with the presence of two small clusters (Figure 3; Table 2) in the control subjects. FM patients with normal biopsy and proximal denervation showed the presence of large clusters with predominant synergy. In IENF-FMN patients, the clusters were located in the time windows that overlapped with the N2 and P2 components (cluster mass = 6.65, cluster size = 1169). For the IENF-FMP subgroup, there were several temporal interactions with one of the largest cluster masses (cluster_mass = 6.88, cluster_size = 1529). The IENF-FMD subgroup showed the presence of few clusters, but with significantly negative values (cluster_mass = −2.27, cluster size = 95), reflecting the redundancy of temporal information between neuronal responses in relation to perceived pain.

### 3.2 Between subjects comparison

A between-group analysis revealed significant differences in TII between the control group and various subgroups of FM patients. The results show that TII values differ between cohorts and vary according to body region and denervation type. The observed differences are often associated with the N2 and P2 components, which serve to highlight specific changes in pain processing mechanisms. In the between-group comparisons, the colormaps represent t-statistics, with positive values indicating higher TII in controls compared to the patient subgroup, and negative values indicating lower TII in controls. Significant clusters (p < 0.05) identified through the cluster-based permutation test are marked separately (e.g., by black contours).

**FIGURE 4:**
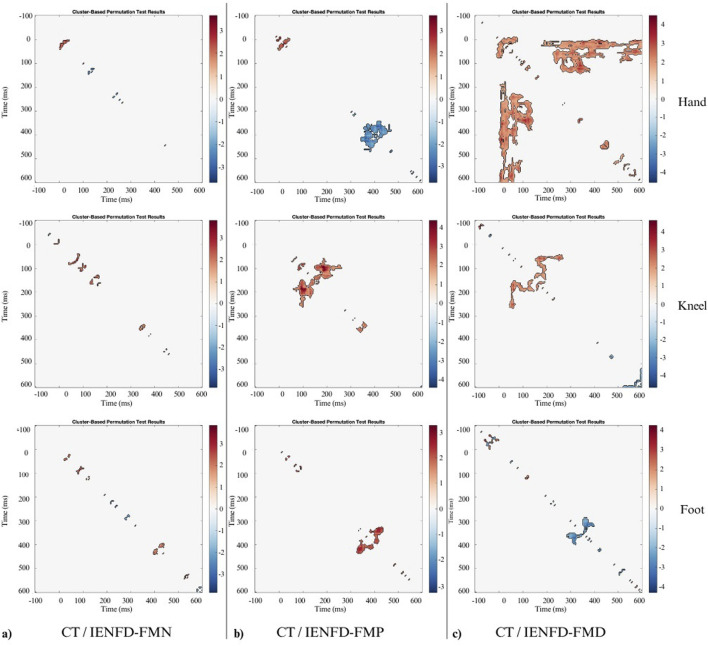
Nonparametric cluster-based permutation analysis of Temporal Interaction Information with statistical significance of 0.05 between: **(a)** CT and FM subgroup with normal biopsy findings (IENFD-FMN); **(b)** CT and FM subgroup with proximal denervation (IENFD-FMP); **(c)** CT and FM subgroup with both proximal and distal denervation (IENFD-FMD). The colormap represents t-statistics for between-group comparisons. Positive values indicate higher TII in controls relative to the patient subgroup; negative values indicate lower TII in controls. Significant clusters (p < 0.05) identified through the permutation test are outlined in black.

A significant cluster was identified in the foot area when the control group was compared with the IENFD-FMN subgroup (Table 3; Figure 4a). This cluster (t1min 395 ms, t1max 410 ms; t2min 418 ms, t2max 438 ms) coincides with the P2 peak and is characterized by a higher TII in controls than in patients with normal skin biopsy (cluster mass = 38.42; cluster size = 20).

**TABLE 3:** Clusters size and weight of the nonparametric cluster-based permutation analysis between controls and FM subgroups.

	District	T1_min (ms)	T1_max (ms)	T2_min (ms)	T2_max (ms)	Cluster_mass	Cluster_size
Ct/IENFD-FMN	Foot	395	410	418	438	38,42	20
Ct/IENFD-FMP	Hand	344	414	368	469	−445,7	207
		−6	17	17	44	56,75	27
	Kneel	149	262	59	145	644,7	269
	Foot	379	434	325	395	225,07	109
Ct/IENFD-FMD	Hand	1	141	176	598	3119,7	1456
		194,5	95	−10	20	9	75
	Kneel	44	145	149	266	443,63	208
	Foot	286	352	352	399	214,44	91

In comparison to the IENFD-FMP subgroup, several clusters were identified in the hand, knee and foot districts (Table 3; Figure 4b). In the hand district, a negative cluster (cluster_mass = −445.7; cluster_size = 207) was observed between 344 and 414 ms for T1 and between 368 and 469 ms for T2, close to the P2 peak. This cluster indicates that patients with proximal denervation have higher T2 values than controls, suggesting a stronger correlation between neuronal activity in these specific time intervals and pain perception. In the knee region, a large positive cluster (cluster_mass = 644.7; cluster_size = 269) was identified between 149 and 262 ms for T1 and between 59 and 145 ms for T2. This indicates that TII is significantly higher in controls than in patients with proximal denervation, which is associated with the peak value of P2.

A comparison between the control group and the IENFD-FMD subgroup showed that the control group had higher TII values in the hand area (Table 3; Figure 4c). This was evidenced by a particularly large cluster (cluster_mass = 3119.7; cluster_size = 1456) between 1 and 141 ms for T1 and between 176 and 598 ms for T2. This cluster included both the N2 and P2 peaks. This result suggests that the control group has a better ability to integrate temporal information compared to patients with diffuse denervation. In addition, significant clusters were identified in the knee and foot districts, albeit of smaller magnitude than in the hand (cluster_mass = 443.63 and 214.44, respectively), consistent with the N2 and P2 peaks.

## 4 Discussion

In this study, we applied a novel analysis of event-related responses, i.e., interaction information, to reveal the interdependence between the vertex complex of laser-evoked responses, the N2-P2 component, and subjective pain perception in control subjects and in patients with fibromyalgia. The relationship is particularly complex because the individual assessment of stimulation intensity could depend on the integrity of the afferents, in this case the a-delta fibers, responsible for the unit of afferent input at the cortical level, as well as on the degree of salience of the painful stimulus (Moayedi et al., 2015). In FM patients, the combination of peripheral small fiber neuropathy and central sensitization phenomena could influence the final subjective report of pain intensity and the nature of the laser-evoked responses, especially the later ones, which are influenced by cognitive factors and stimulus relevance. In the present analysis, we found that pain ratings appeared to be elevated across patients, but there was high variability within FM groups, even when the skin over the knee - a tender point - was stimulated, confirming the complex mechanism underlying the subjective feeling of the intensity of a noxious stimulus. This complexity reflects the broader principle that physiologic function emerges from temporally structured interactions, a view supported by models of transient network topology shifts described in Network Physiology (Bashan et al., 2012).

Thus, our experiment was based on the hypothesis that the common mutual information expressed by the temporal representation of vertex LEPs, and subjective pain sensation could produce different patterns in controls and FM patients, taking into account the presence and distribution of small fiber neuropathy.

Although the definitions of synergy and redundancy originate from information theory, we propose a physiological interpretation in line with previous EEG applications of TII (Ravijts, 2019). In our context, synergy may reflect temporally distributed cortical interactions that jointly contribute to pain encoding, while redundancy may indicate a less selective, potentially maladaptive persistence of nociceptive activity. Given the novelty of this approach in clinical populations, these interpretations should be considered as translational hypotheses, albeit consistent with existing neurophysiological literature.

We observed that clusters of synergy between the time course and pain ratings occurred in correspondence of N2 and P2 peaks for hand stimulation in controls and patients with normal skin biopsy and proximal denervation, indicating a correspondence between the temporal characteristics of late LEPs and subjective pain perception in these groups, a correlation that was not observed for the amplitude of the same peaks.In FM patients, especially those with proximal denervation who theoretically could not include distal upper limb sites, the synergy was even stronger than in controls, although pain ratings in the hand were only slightly increased compared to non-FM cases. Thus, the interaction information could express an integrated pattern of processing noxious stimuli that is independent of the individual pain rating values, or the characteristics of the cortical responses associated with nociceptive afferents. In patients with distal denervation, synergy clusters were weaker compared to controls. Although we did not measure the density of intraepidermal nerve fibers in the hand, this is a distal site that is frequently denervated in FM patients (De Tommaso et al., 2014).Thus, we can assume that denervation is predominant at distal sites in FMD patients, so that the interaction information pattern could express a dysfunctional mode of pain processing for a possible mismatch between cortical activation and subjective stimulus intensity assessment due to a tendency to temporal dispersion of LEPs for the partial impairment of afferent volley.

From a systems-level perspective, the temporal synergy and redundancy patterns that were observed may be indicative of adaptive or dysfunctional network reconfigurations. It is proposed that the presence of widespread redundancy in patients with distal denervation could indicate a loss of temporal specificity in pain processing. This interpretation is corroborated by the findings of brain wave interaction studies, which indicated that dynamic flexibility in temporal coupling serves as an indicator of physiological adaptability (Liu et al., 2015). The TII framework may thus offer a complementary approach to mapping such flexibility in pathological conditions, in line with the aims of Network Physiology to characterize the human physiome (Ivanov, 2021).

Looking at the proximal site of the lower limb, there was a synergistic pattern in the correspondence of N2 and P2 waves in patients with normal innervation of the small fibers and distal denervation and in the correspondence of P2 and also after 400 msec in patients with proximal denervation. Control subjects showed a synergy cluster in the correspondence of N2 that was more related to the discriminative quality of painful stimuli, while FM patients tended to activate the cortical areas responsible for the affective component of pain and stimulus-related arousal (Valeriani et al., 2000). The site of stimulation was the knee, which corresponds to a tender point (Wolfe et al., 1990), a site with particular sensitivity to pain in FM patients, who probably tend to make their subjective judgment with a predominant affective content. When comparing between groups, controls showed increased activation in terms of a synergistic cluster between pain ratings and the time course of N2, especially when compared with patients with proximal and distal denervation. The shared mutual information may reflect the complexity of FM, in which subjective pain assessment and associated cortical responses are definitely influenced by the degree of denervation of small fibers, even in the presence of a strong arousal and affective response to noxious stimuli (De Tommaso et al., 2022).

During stimulation of the foot, FM patients with normal skin biopsy and proximal denervation showed clear synergy clusters, which were in any case weaker than in the controls, at least in the patients with proximal denervation even in the time frame of P2. This finding may indicate a dysfunctional pattern related to possible involvement of small nociceptive afferents at the distal sites in some patients. This differed from the pattern observed at the knee, which is a sensitive point that determines a marked arousal and affective response expressed in the late positive component of LEPs. (Vecchio et al., 2022). In patients with distal denervation, the redundant pattern prevailed in the P2 time frame compared to controls, suggesting a loss of additive information between the late time course of LEPs and subjective pain ratings. This was likely related to the dysfunction of the nociceptive a-delta afferents, even in the presence of a slight increase in VAS scores elicited by foot stimulation.

From a methodological perspective, the present study contributes to the growing field of time-resolved network analysis in neurophysiology by introducing TII as a tool to quantify how neural signals jointly encode a behavioural variable across time. Conventional measures of connectivity characterise interactions between spatially distinct channels or regions. In contrast, TII examines second-order dependencies within a single-channel signal across temporal delays. This reveals how past and present neural states combine to form predictive patterns. This is of particular pertinence in the context of EEG, where temporal resolution is high, yet spatial interpretability remains constrained.

Recent advances in temporal interaction modelling, including dynamic recurrent embedding frameworks for temporal graphs (Kumar et al., 2019) and neural network-based encodings of time-sensitive signals (Xia et al., 2022), indicate the necessity of capturing complex, non-linear temporal dependencies in physiological data.

TII aligns with these approaches by providing a computationally tractable yet conceptually rich metric that can detect synergy or redundancy between temporally distant points in the EEG time series. Furthermore, its capacity to localise these effects around physiologically meaningful windows (e.g., the N2–P2 complex) enhances its interpretability within classical ERP frameworks. This positions TII as a complementary tool to both information-theoretic and network-based analyses, suitable for capturing functional dynamics that are often missed by standard univariate or static connectivity measures.

### 4.1 Limitations

The groups considered for the analysis differed in the number of cases, an element that could affect the reliability of the statistical analysis, although this was partially corrected by the cluster permutation test approach adopted. In this pilot study, we considered the Cz channel, commonly used for N2P2 analysis of the vertex, while in some cases other channels above the midline might better express late pain-related cortical responses.

We applied a very novel high-order temporal interaction analysis that reflects the interaction between biological variables in terms of synergy and redundancy. The interpretation of the statistical results might be difficult and to some extent speculative.

### 4.3 Conclusion

When first looking at the higher order temporal interaction analysis of the time course of late LEPs and subjective pain ratings, FM patients with proximal and distal denervation showed more pronounced differences compared to controls in terms of loss of synergy and increased redundancy in vertex complex time frames. This may suggest that small fiber neuropathy in FM patients, although not consistent with the clinical picture (Fasolino et al., 2020) or the main features of LEPs (Vecchio et al., 2020; 2022), could affect cortical activation related to painful stimuli and subjective pain rating in a complex way.

These preliminary results may encourage further application of high-order temporal interaction paradigms in the study of complex chronic pain syndromes.

## Data Availability

The raw data supporting the conclusions of this article will be made available by the authors, without undue reservation.

## Abbreviations

LEPs, Laser Evoked Potentials; VAS, Visual Analogic Scale; FM, Fibromyalgia; II, Interaction Information; TII, Temporal Interaction Information; IENFD, Intraepidermal Nerve Fibers Density; FMN, Fibromyalgia with normal skin biopsy; FMP, Fibromyalgia with proximal denervation; FMD, Fibromyalgia with proximal and distal denervation; MI, Mutual information.
