# Supplementary material for: Temporal interaction information and laser evoked responses: preliminary results in fibromyalgia patients with small fibers pathology
Source: Front Netw Physiol. 2025 Sep 1;5:1592518. doi: 10.3389/fnetp.2025.1592518 (PMC12433961; doi:10.3389/fnetp.2025.1592518)
Supplement: Supplementary file 1 [file Supplementaryfile1.docx]

**Supplementary materials**

***Stimulation procedure***

Laser pulses (wavelength, 10.6 mm; beam diameter, 2 mm; duration of stimulus 30 ms) generated by a CO₂ laser (Neurolas Electronic Engineering, Florence, Italy) were used to induce pain. (see supplementary material) A total of 30 pulses were administered at each stimulation site, with the intensity set at 1.5 W above the individual pain threshold and a 10-second interval between each pulse. To exclude possible injury to the skin, fatigue or sensitization of the nociceptors, the position of the laser beam was changed after each pulse. A five-minute interval was maintained between each series of stimuli. Stimulation was applied randomly to the dorsal surface of the right hand, the distal thigh (20 cm below the iliac spine) and the dorsal surface of the foot. The proximal and distal sites on the leg were selected based on the results of the skin biopsies. Subjects were instructed to focus on the intensity of the stimulus, and a color scale ranging from 0 (white, indicating no pain) to 100 (red, indicating the most intense pain) was provided to facilitate pain rating. After the administration of each series of stimuli, patients were asked to rate their perceived pain intensity using a visual analog scale (VAS) from 0 to 100. Stimuli were applied to the back of the right hand, the skin region above the knee and the back of the right foot.

**Recording procedure**

As reported in Vecchio et al. (2020), an assembly of 65 scalp electrodes, referred to as Nasion, was used. The recording device was Micromed Brain Quick. The EEG data were digitized at 256 Hz. Details can be found in the supplementary material. Two additional electrodes were placed above the eyebrows to record the electrooculogram (EOG). In addition, the ground electrode was located in the frontopolar zone.

***Processing***

The preprocessing was performed with MATLAB using the tool EEGLAB 14_1_1. At the beginning of the process, the data was subjected to a top filtering procedure with a frequency of 1 Hz to eliminate slow drifts. Subsequently, a notch filter was applied at 50 Hz (lower limit: 48 Hz, upper limit: 52 Hz) to remove artefacts caused by the noise of the power lines. To perform preliminary calculations of the channel measurements, the independent component analysis (ICA) components associated with eye artefacts (EOG) were removed and the missing channels were interpolated using a spherical method. Problematic channels were identified by applying a semi-automated method based on visual analysis and examination of channel statistics. Channels whose distributions of potential values deviated significantly from a Gaussian distribution were eliminated.The Cz channel was included in all cases except 2 FM cases, where the presence of motion artifacts in at least one of the stimulation sites led to exclusion from the final analysis. The vertex-N2P2 complex was calculated by considering a time window of 800 ms after the stimulus, applying a low-pass filter at 70 Hz, and removing the baseline by considering the 100 ms before the laser stimulus. For both the patient and control groups, data from 30 trials were averaged for each stimulation site and the overall average across all cases was calculated for each group and used to calculate the information-theoretic quantity.

**Skin biopsy**

The method of skin biopsy was described in detail in (Vecchio et al., 2020) and the details can be found in the supplementary material. In all patients, 3 mm punch biopsies were taken from the thigh and distal leg after an intradermal injection of 1% xylocaine. The samples were fixed in 2% paraformaldehyde lysine sodium periodate at 4°C overnight. They were then cryopreserved, serially sectioned with a cryostat and immunostained with polyclonal anti-protein gene product 9.5 (Ultraclone Ltd). We calculated intraepidermal nerve fiber density on three non-consecutive central sections by brightfield microscopy using a stereology workstation (Olympus BX50, PlanApo oil objective 40x/NA = 1.0) and compared it with sex- and age-adjusted normal values (Devigili et al., 2008; Lauria et al., 2010). Based on a consistent difference from normative data (Lauria et al, 2010) of more than 2 SD, patients were categorized into those with proximally reduced IENFD-FMP, distally and proximally reduced IENFD-FMP, and those with normal IENFD-FMD.
